# UPDATES ON THE TREATMENT OF ANTERIOR TIBIAL TUBEROSITY AVULSION FRACTURES IN ADOLESCENTS

**DOI:** 10.1590/1413-785220263403e296371

**Published:** 2026-06-22

**Authors:** Flávia Rachel Nogueira de Negreiros Freitas, Carla Mariana Gonçalves Carvalho e Silva, Danilo do Nascimento Viana, José Alves da Silva, Alexandre Almeida Borges, Osvaldo Campos Pereira

**Affiliations:** 1 Universidade Estadual do Piaui (UESPI), Departamento de Ortopedia e Traumatologia, Teresina, PI, Brazil.

**Keywords:** Fractures, Avulsion, Tibia, Adolescent, Treatment Outcome, Fratura Avulsão, Tíbia, Adolescente, Resultado do Tratamento

## Abstract

Anterior tibial tuberosity avulsion fracture (ATF) is a rare injury in adolescents, usually associated with sports activities, ranging from mild to complex cases. Early diagnosis and appropriate treatment are essential to recover knee function and avoid complications. This study analyzed updates and innovations in the treatment of ATF in adolescents, highlighting recent therapeutic approaches and their efficacy. A narrative review of the literature was performed in the Lilacs, PubMed and Capes Periodicals databases. Treatment should be individualized, with a conservative approach for stable fractures and surgical fixation for severe cases. Advances such as fluoroscopy-guided percutaneous fixation have optimized recovery, allowing better joint assessment and less invasiveness. Postoperative management emphasizes early mobilization and physiotherapeutic rehabilitation, favoring functional recovery and a safe return to sports. The prognosis is generally favorable, provided the diagnosis is accurate and the follow-up is rigorous. Rehabilitation is essential to restore muscle strength and prevent relapses. Despite advances, the scarcity of long-term studies highlights the need for more research to standardize protocols and improve clinical outcomes. ***Level of Evidence II, Review Study.***

## RESUMO

A fratura-avulsão da tuberosidade anterior da tíbia (FATAT) é uma lesão rara em adolescentes, geralmente associada a atividades esportivas, variando de casos leves a complexos. O diagnóstico precoce e o tratamento adequado são fundamentais para recuperar a função do joelho e evitar complicações. Este estudo analisou as atualizações e inovações no tratamento da FATAT em adolescentes, destacando abordagens terapêuticas recentes e sua eficácia. Foi realizada uma revisão narrativa da literatura nas bases Lilacs, PubMed e Periódicos Capes. O tratamento deve ser individualizado, com abordagem conservadora para fraturas estáveis e fixação cirúrgica para casos graves. Avanços como a fixação percutânea guiada por fluoroscopia têm otimizado a recuperação, permitindo melhor avaliação articular e menor invasividade. O manejo pós-operatório enfatiza mobilização precoce e reabilitação fisioterapêutica, favorecendo a recuperação funcional e o retorno seguro ao esporte. O prognóstico é geralmente favorável, desde que o diagnóstico seja preciso e o acompanhamento rigoroso. A reabilitação é essencial para restaurar a força muscular e prevenir recidivas. Apesar dos avanços, a escassez de estudos de longo prazo evidencia a necessidade de mais pesquisas para padronizar protocolos e aprimorar os resultados clínicos. ***Nível de Evidência II, Estudo de Revisão.***

## INTRODUCTION

Anterior tibial tuberosity avulsion fractures (ATF) are rare but clinically significant injuries that predominantly occur in adolescents during the skeletal growth phase. These fractures are characterized by the displacement of the bony fragment from the tibial tuberosity due to excessive traction from the patellar tendon, usually during high-intensity physical activities such as jumping or running. The incidence is higher in boys aged 14 to 16, coinciding with the period of closure of the proximal tibial physis, which makes the area more vulnerable to traction forces.^1^

ATF occurs when the tension exerted by the patellar ligament exceeds the combined resistance of the underlying physes, the surrounding perichondrium, and the adjacent periosteum. This injury can result from two main mechanisms: abrupt and intense contraction of the quadriceps muscle against a fixed tibia, usually observed during explosive jumps, or sudden passive flexion of the knee while the quadriceps remains contracted. Additionally, associated injuries may occur that affect the surrounding ligaments, the menisci, and, in rare cases, the tibial plateau.^2^

It is also noteworthy that ATF is frequently associated with pre-existing conditions, such as Osgood-Schlatter disease, which can weaken the patellar tendon's insertion at the tibial tuberosity. Although the relationship between these conditions is not completely causal, the presence of Osgood-Schlatter may increase the risk of avulsion during sports activities.^3^ Furthermore, the classification of these fractures has evolved over the years, with contributions from Watson-Jones, Ogden, Tross, and Murphy, and more recently by Ryu and Debenham, who proposed the inclusion of type IV fractures, involving the proximal epiphysis of the tibia and the posterior metaphysis.^1,4^

The treatment of ATF depends on the classification of the fracture, the extent of displacement, and the presence of complications, such as compartment syndrome. Less severe fractures (types IA, IB, and IIA) can be treated conservatively with immobilization and load restriction, while more complex fractures (types III, IV-A, and IV-B) require open reduction and internal fixation with cannulated screws to ensure stability and proper consolidation.^4^

Post-operative rehabilitation plays a crucial role in functional recovery, with intensive programs aimed at restoring range of motion, muscle strength, and the ability to return to daily and sports activities. Recent studies highlight that, with appropriate treatment, most patients achieve excellent functional outcomes, with a low impact on quality of life.^5^

Given the clinical relevance of ATF and the potential complications associated with inadequate diagnosis and treatment, it becomes essential to conduct studies that systematize and critically analyze the available therapeutic strategies. The evolution of surgical techniques, combined with the improvement of rehabilitation protocols, reinforces the need for updated reviews that guide clinical decision-making. Thus, this study is justified by the importance of understanding innovations in the management of these injuries, contributing to the optimization of functional outcomes and the improvement of the quality of life of affected adolescents.

## METHODOLOGY

This research consists of a narrative review of the literature, a suitable format for describing and examining the evolution of a specific topic. As it is a qualitative approach, it provides the reader with the opportunity to deepen and update their knowledge on the subject. This type of study does not require a methodology that allows for data reproduction or quantitative analysis.^6^

### Research strategy in databases

The narrative review on the treatment of avulsion fractures of the anterior tibial tuberosity was developed through research in the databases Latin American and Caribbean Literature in Health Sciences (LILACS), *Scientific Electronic Library Online* (SciELO), and PubMed. The study was conducted in January and February 2025. For the selection of articles, the descriptors "*Avulsion Fractures of the Anterior Tibial Tuberosity", "Adolescent"* were combined with the boolean operator "AND."

### Eligibility criteria

The established inclusion criteria were articles published in Portuguese and English that had a direct relationship with the topic addressed and that had been published in the period from 2018 to 2024. In contrast, the exclusion criteria included studies that were not aligned with the proposed subject, publications in languages other than those specified, and articles that were only partially available.

### Data organization

After the search stage, the articles were classified and structured according to the main themes, being organized by areas of knowledge. During the analysis, the publications chosen to compose the review were segmented into four central axes, which will be explored throughout the study, namely:

1. Definition, Etiology, and Classification of Anterior Tibial Tuberosity Avulsion Fractures;
2. Diagnostic Methods and Clinical Assessment;
3. Conventional Treatments and Current Approaches;
4. Innovations and Future Trends in Fracture Management.

### Selection of studies

**Figure 1:**
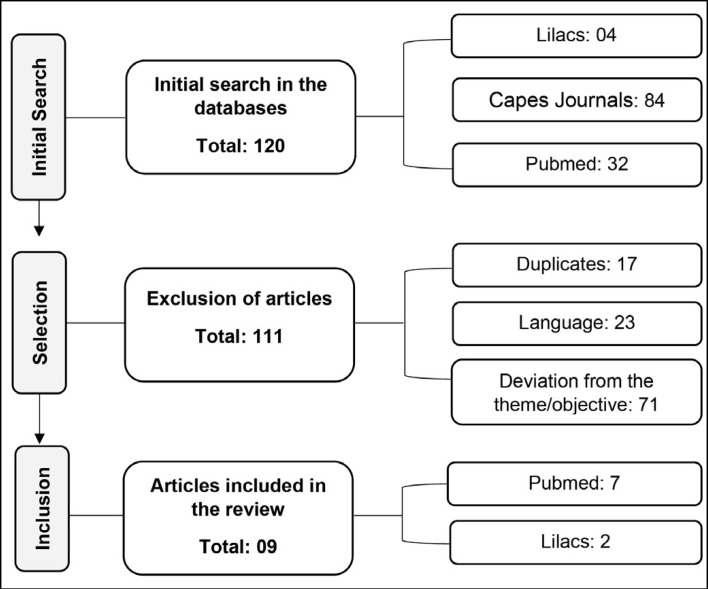
Review and selection of the articles included in the review.

Source: Author (2025).

Initially, a total of 120 articles were located in the selected databases, with 4 from Lilacs, 32 from PubMed, and 84 in the Capes Periodicals Portal. Based on the eligibility criteria, 111 articles were excluded, leaving 9 for review and inclusion in the narrative review (Figure 1).

## RESULTS AND DISCUSSION

The results of this narrative review explore fundamental aspects of ATF, covering its definition, etiology, and classification, in addition to the main diagnostic methods used in clinical practice. The analysis includes a review of conventional therapeutic approaches, from conservative treatment to surgical techniques indicated for different degrees of injury severity. Furthermore, recent innovations in the management of these fractures are discussed, focusing on advances in bone fixation, new rehabilitation strategies, and future perspectives to optimize clinical and functional outcomes for patients.

Nine studies were highlighted, most of which addressed adolescents aged 14 to 15 years, with ages ranging from 12 to 18 years. All participants were male, and the use of cannulated screw fixation was emphasized as a prior technique.

### General aspects of anterior tibial tuberosity avulsion fractures

The tibial tuberosity originates from a secondary ossification center located in the proximal portion of the tibia. Unlike the proximal epiphysis of the tibia, which forms under compressive forces, the tibial tuberosity is classified as an apophysis and develops under tension. The process of tubercle formation is divided into four stages: cartilaginous, apophyseal, epiphyseal, and osseous. The closure of the proximal epiphysis of the tibia occurs progressively, advancing distally towards the tuberosity apophysis, creating a period of greater mechanical vulnerability, which may favor the occurrence of avulsion fractures. This characteristic partly explains the rarity of recurrences of this type of injury, although they may occur, especially in pre-adolescent patients aged between 9 and 12 years.^4^

According to Kunis et al.^7^, the ATF are traumatic injuries that predominantly occur in adolescents, characterized by the displacement of the bone fragment from the tibial tuberosity due to excessive traction of the patellar tendon. This region is particularly vulnerable during the skeletal growth period, when the proximal tibial physis is in the process of closing. The ATF is considered a rare injury, with reported incidence between 0.4% and 2.7% of pediatric fractures, being more common in boys aged between 14 and 16 years.

The etiology of ATF is directly related to mechanisms of indirect trauma, such as the violent contraction of the quadriceps muscle against a fixed foot, common in sports activities involving jumps, such as basketball and soccer. Another frequent mechanism is the abrupt flexion of the knee against a vigorous contraction of the quadriceps, as occurs during the landing of a jump.^2^

Predisposing factors include the presence of conditions such as Osgood-Schlatter disease, which weakens the insertion of the patellar tendon at the tibial tuberosity, increasing the risk of avulsion during high-intensity activities.^3^ Additionally, the developmental phase of the tibial tuberosity, which occurs under tension (in contrast to the proximal tibial epiphysis, which develops under compression), makes the region more susceptible to injuries during the closure period of the physis.^7^

The classification of ATF has evolved over the years, with significant contributions from various authors. The original classification by Watson-Jones^8^ describes three types of fractures:

- Type I: Avulsion of a small portion of the tibial tuberosity, distal to the proximal physis.
- Type II: Fracture that extends through the physis but does not involve the knee joint.
- Type III: Fracture that extends proximally to the physis, involving the knee joint.

**Figure 2:**
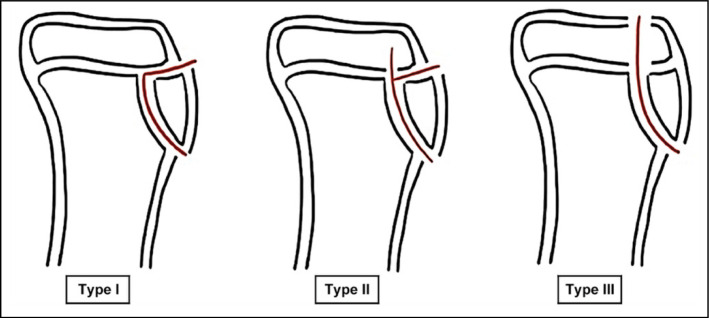
Watson-Jones classification system.

Source: Adapted from Kunis et al. (2021).

Figure 2 presents the Watson-Jones classification (1974), which categorizes fractures into three types: I, II, and III.

Subsequently, Ogden et al.^9^ expanded this classification to include subtypes (A and B) that consider the degree of displacement and comminution, which explain: IA: fracture through the center of ossification, without displacement; IIB: fracture through the center of ossification, with minimal displacement; IIA: separation of the anterior tuberosity of the tibia; IIB: comminution of the center of ossification; IIIA: trans-epiphyseal articular fracture without comminution; IIIB: trans-epiphyseal articular fracture with comminution. Ryu and Debenham^10^ proposed the addition of Type IV, which involves the avulsion of the proximal tibial epiphysis and the posterior metaphysis, creating a more complex configuration.

More recently, Frankl et al.^11^ suggested the inclusion of a Type C, which encompasses fractures associated with the avulsion of the patellar ligaments, while McKoy and Stanitski^12^ proposed Type V, characterized by a "Y" fracture that combines elements of Types III and IV.

This evolution in classification reflects the anatomical complexity and diversity of fracture patterns, allowing for a more precise and individualized therapeutic approach.^7^

### Diagnostic methods and clinical assessment

The diagnosis of ATF is based on a combination of clinical assessment and imaging, aimed at identifying the extent of injury, the degree of displacement, and possible associated complications.^4^ The clinical presentation of ATF is generally acute, with a history of indirect trauma during sports activities, such as jumping or running. Patients often report intense pain in the anterior region of the knee, accompanied by local swelling and an inability to actively extend the leg. On physical examination, visible deformity may be observed at the tibial tuberosity, tenderness to palpation, and, in severe cases, an inability to ambulate.^13^

The conventional assessment of avulsion fractures of the anterior tibial tuberosity is performed through radiographs in anteroposterior (AP) and lateral views of the knee. Since the tibial tuberosity is not perfectly aligned with the midline of the tibia, a slight internal rotation of the leg can position it perpendicular to the radiographic film, providing a more accurate view of the injury. Additionally, the presence of a high patella can be identified in lateral images, aiding in the analysis of the clinical picture.^14^

According to Zaizi et al.^15^, plain radiography is the initial method of choice, while Computed Tomography (CT) and Magnetic Resonance Imaging (MRI) are reserved for complex cases or when there is suspicion of associated injuries. Early and accurate identification of the fracture is crucial to guide treatment and prevent complications, such as compartment syndrome or loss of knee function.

### Conventional treatments and current approaches

The treatment of ATF is determined based on the classification of the fracture, the degree of displacement, and the presence of associated complications. Therapeutic approaches range from conservative methods to surgical interventions, with the main objective of restoring the integrity of the extensor mechanism and, when necessary, preserving the articular surface.^4^

Fractures with minimal displacement (types IA, IB, and IIA) can be treated conservatively. Treatment includes immobilization with a cast or brace in full extension of the knee for four to six weeks, followed by progressive rehabilitation. Load restriction is recommended until there is radiographic evidence of bone consolidation.^14^

The conservative approach is primarily indicated for patients with stable fractures and no compromise of the knee joint. However, it is essential to regularly monitor fracture alignment and consolidation with serial X-rays to avoid complications such as nonunion or loss of reduction.^14^

In less severe cases, conservative treatment involves closed reduction of the fracture, followed by immobilization with a long or cylindrical cast for approximately four weeks until bone consolidation is confirmed on imaging. Fractures with greater displacement or joint involvement require open reduction with internal fixation, using screws, tension band wires, Kirschner wires, Steinmann pins, or periosteal sutures, followed by a period of immobilization of three to four weeks.^4^

Fractures with significant displacement (types III, IV-A, IV-B, and V) or involvement of the knee joint generally require RAFI. RAFI allows for direct visualization of the fracture, ensuring anatomical reduction and stable fixation. Cannulated screws are often used to fix the fractured fragment, while plates and Kirschner wires may be employed in more complex cases.^15^

Regarding the treatment of associated complications, it is noteworthy that compartment syndrome is a potentially serious complication of ATF and requires immediate diagnosis and treatment. Fasciotomy is the procedure of choice to relieve intracompartmental pressure and prevent irreversible damage to soft tissues.^16,17^

Furthermore, the importance of postoperative rehabilitation is emphasized, as it is a critical component of ATF treatment, regardless of the chosen approach. Physical therapy programs are initiated early to restore the range of motion, muscle strength, and knee function. Partial weight-bearing is gradually allowed, with a full return to sports activities after 3 to 6 months, depending on clinical and radiographic progress.^1,18,19^

Postoperative management generally follows protocols that prioritize initial immobilization, early weight support, and gradual progression of range of motion. Symptomatic pain control and physiotherapeutic rehabilitation are essential for the recovery of muscle strength and joint stability. The choice of treatment should be individualized, taking into account the specific characteristics of the fracture and the patient. The advancement of minimally invasive techniques and the development of bioabsorbable materials have contributed to better functional outcomes and a lower incidence of complications, demonstrating significant progress in the treatment of ATF.^7^

### Therapeutic approaches used in recent studies

In recent years, various therapeutic approaches have been explored and refined in the management of tibial tuberosity avulsion fracture, a rare but significant injury that primarily affects adolescents during sports activities. Recent studies highlight advances in both conservative treatments, indicated for minimally displaced cases, and surgical treatments, recommended for unstable fractures or with significant displacement. Additionally, new fixation techniques, such as cannulated screws and tension wires, have been developed to improve fracture stability and accelerate rehabilitation. The personalization of treatment, considering factors such as fracture pattern, patient skeletal maturity, and functional demand, has become an essential principle in clinical decision-making. In this way, the analysis of current therapeutic strategies allows for understanding their efficacy and safety, as well as identifying trends and gaps that require further investigation to improve specific outcomes and prevent complications.

In this perspective, the study by Pacífico Júnior et al.^1^ reports the case of an adolescent with an avulsion fracture of the tibial tuberosity, where the diagnosis was confirmed by imaging tests, and the treatment performed was surgical fixation with screws, which showed good results regarding the treatment used, with adequate stabilization of the bone fragment and allowed for progressive rehabilitation. The postoperative follow-up reported good clinical evolution, with bone lesions recovered and complete functional recovery of the knee, reinforcing the efficacy of surgical treatment in this condition.

Zhao et al.^3^ reported a case of an adolescent with an avulsion fracture of the anterior tibial tuberosity associated with Osgood-Schlatter disease, a condition that weakens the patellar tendon insertion and increases susceptibility to this type of injury. The patient presented with intense pain and swelling in the knee after a sudden contraction of the quadriceps during a sports activity, and the diagnosis was confirmed by imaging tests, which revealed the displacement of the bone fragment. The treatment involved open reduction under epidural anesthesia, surgical fixation with cannulated screws, and reinforcement with sutures, ensuring stabilization of the fracture. After two weeks, the stitches were removed, and healing was completed, with the knee kept immobilized for four weeks before starting exercises for restoring range of motion and muscle strengthening. At the three-month follow-up, joint mobility had already reached 0° to 120°, and the implants were removed after 12 months, allowing the patient to resume activities without pain or functional limitations. In the study by Pedrazzini et al.^18^, a 13-year-old adolescent suffered a ATF during a basketball game, presenting with intense pain and inability for active extension of the knee. The diagnosis was confirmed by X-rays and CT scans, which revealed lateral displacement of the patella and possible injury to the lateral ligamentous retinacula. Classified as Type IB according to Ogden, the fracture was treated with RAFI using cannulated screws to ensure anatomical restoration and mechanical stability. The procedure was performed 10 days after the trauma, through an anterior approach with an incision centered on the tibial tuberosity. The postoperative period followed a structured rehabilitation protocol, beginning with physical therapy to restore joint mobility, followed by muscle-strengthening exercises. After three months, the patient showed no pain, limping, or local tenderness, being cleared for a progressive return to sports activities, including basketball, without complications or functional sequelae. Similarly, in the case report by Zaizi et al.^15^, an adolescent suffered an ATF during a school sports activity, characterizing a mechanism of injury associated with sudden and intense contraction of the quadriceps muscle. The diagnosis by imaging tests revealed the displacement of the bone fragment. Given the fracture pattern and degree of displacement, surgical treatment was chosen using the RAFI technique with cannulated screws, aiming for anatomical restoration and stability of the extensor mechanism. In the postoperative period, the patient followed a protocol of temporary immobilization, followed by progressive physical therapy to restore mobility and muscle strength. The evolution was satisfactory, with adequate bone consolidation, absence of complications, and complete functional return to sports activities after rehabilitation.

In addition to these, in the study by Giunchi et al.^19^, the authors described two rare cases of simultaneous bilateral avulsion of the anterior tibial tuberosity in adolescents occurring during sports activities. Both patients presented with severe pain, swelling, and inability to actively extend their knees after jumping during basketball practice. The diagnosis was confirmed by X-rays and CT scans, which revealed avulsion fractures classified as Type III according to Watson-Jones. The treatment consisted of RAFI with cannulated screws in both knees, performed in a single surgical session. The fixation was supplemented with absorbable sutures to reinforce the insertion of the patellar tendon. Postoperatively, the patients were immobilized with braces and kept non-weight bearing for 6 weeks, followed by a progressive rehabilitation protocol. After 3 months, both patients showed complete radiographic consolidation, recovery of range of motion, and return to sports activities without complications.

In the study by Bombah et al.^14^, the authors reported two cases of ATF among Cameroonian adolescents occurring during sports activities. Both patients were boys and sustained the injury after a violent contraction of the quadriceps during sports (high jump and soccer). The fractures were classified as Type IV according to Ryu and Debenham (case 1) and Type IA according to Ogden (case 2). The treatment for the first case was surgical, with internal fixation through double screw fixation, while the second case was treated conservatively, with immobilization using a cast brace. Both patients achieved excellent functional outcomes, with complete fracture consolidation, recovery of range of motion, and return to sports activities without complications.

Therefore, it is emphasized that these cases highlight the importance of an individualized approach, considering the classification of the fracture and the degree of displacement, and reinforce the effectiveness of both surgical and conservative treatments, when appropriately indicated, to ensure good clinical and functional results.

## CONCLUSION

According to the analyzed data, it was found that simultaneous bilateral avulsion of the anterior tubercle of the tibia in adolescents is a rare condition, with a lack of consensus on the ideal therapy. The management of these fractures should be individualized, taking into account the severity of the injury, the degree of bone displacement, and the presence of associated injuries. Early diagnosis and appropriate treatment choice, whether conservative or surgical, are essential to ensure effective recovery and minimize complications. Surgical approaches, such as internal fixation with cannulated screws, have demonstrated effectiveness in restoring extensor mechanism stability, allowing a gradual return to functional activities. Furthermore, postoperative rehabilitation is crucial for restoring muscle strength and preventing recurrences. Despite advances in treatment, the lack of long-term studies on this condition highlights the need for further research to establish more standardized protocols and improve clinical outcomes.

## Data Availability

The authors confirm that all data supporting the findings of this study are available within the article.
